# Age of first exposure to soccer heading: Associations with cognitive, clinical, and imaging outcomes in the Einstein Soccer Study

**DOI:** 10.3389/fneur.2023.1042707

**Published:** 2023-02-09

**Authors:** Molly F. Charney, Kenny Q. Ye, Roman Fleysher, Bluyé DeMessie, Walter F. Stewart, Molly E. Zimmerman, Mimi Kim, Richard B. Lipton, Michael L. Lipton

**Affiliations:** ^1^Gruss Magnetic Resonance Imaging Center, Albert Einstein College of Medicine and Montefiore Medical Center, Bronx, NY, United States; ^2^Department of Radiology, Albert Einstein College of Medicine and Montefiore Medical Center, Bronx, NY, United States; ^3^Department of Epidemiology and Population Health, Albert Einstein College of Medicine, Bronx, NY, United States; ^4^Department of Systems and Computational Biology, Albert Einstein College of Medicine, Bronx, NY, United States; ^5^Medcurio Inc., Oakland, CA, United States; ^6^Department of Psychology, Fordham University, Bronx, NY, United States; ^7^Saul B. Korey Department of Neurology, Albert Einstein College of Medicine, Bronx, NY, United States; ^8^Department of Neurology, Montefiore Medical Center, Bronx, NY, United States; ^9^Department of Psychiatry and Behavioral Sciences, Albert Einstein College of Medicine and Montefiore Medical Center, Bronx, NY, United States; ^10^Dominick P. Purpura Department of Neuroscience, Albert Einstein College of Medicine and Montefiore Medical Center, Bronx, NY, United States

**Keywords:** soccer, heading, DTI, NODDI, cognition, imaging, repetitive head impact (RHI)

## Abstract

**Introduction:**

The objective of this study is to assess the role of age at first exposure (AFE) to soccer heading as a predictor of known adverse associations of recent and longer-term heading with brain microstructure, cognitive, and behavioral features among adult amateur soccer players.

**Methods:**

The sample included 276 active amateur soccer players (196 male and 81 female) aged 18–53 years old. AFE to soccer heading was treated as a binary variable, dichotomized at ≤ 10 years vs. >10 years old, based on a recently promulgated US Soccer policy, which bans heading for athletes ages 10 and under.

**Results:**

We found that soccer players who began heading at age 10 or younger performed better on tests of working memory (*p* = 0.03) and verbal learning (*p* = 0.02), while accounting for duration of heading exposure, education, sex, and verbal intelligence. No difference in brain microstructure or behavioral measures was observed between the two exposure groups.

**Discussion:**

The findings indicate that, among adult amateur soccer players, AFE to heading before age 10 compared to later start of heading, is not associated with adverse outcomes, and may be associated with better cognitive performance in young adulthood. Cumulative heading exposure across the lifespan, rather than early life exposure, may drive risk for adverse effects and should be the focus of future longitudinal studies to inform approaches to enhance player safety.

## 1. Introduction

Long-term adverse neurologic consequences of sport-related repetitive head impacts (RHI) have been evident since the description of *Dementia pugilistica* more than 100 years ago (1). Recent studies of contact sport athletes have identified adverse effects of RHI on cognition, brain microstructure, and brain metabolism (2–8). With over 265 million players worldwide, adverse consequences of RHI in soccer could pose a major public health challenge (9). RHI in soccer typically occur due to heading of the soccer ball as well as collisions and falls. Multiple reports have described adverse immediate, recent, and longer-term adverse CNS effects of heading as measured by neuroimaging and cognitive assessment. Studies have also identified heading as a greater contributor to worse cognitive performance than recognized concussion (10–12).

Childhood exposure to RHI is an area of concern expressed by researchers, clinicians, sport organizations, and the lay public, both for its immediate effects during childhood and out of concern it could lead to later life neurological dysfunction and neurodegeneration. Reasons for this concern include the potentially greater vulnerability of the developing brain to RHI and the assumption that earlier onset of exposure to RHI equates with greater overall lifetime exposure. Rule changes have been implemented to limit or exclude RHI at younger ages in American style football, ice hockey and soccer, but little evidence supports limitations based on the age at first exposure (AFE) to RHI.

AFE to American style football—a proxy for AFE to RHI—has been used in multiple studies to assess effects of early exposure to RHI. AFE has been treated as a binary variable, based on a cutoff of 12 years of age, in samples of American football, soccer, ice hockey, and lacrosse athletes, and competitive fighters. The *a priori* choice of age 12 as the AFE cutoff has been based on a critical period of brain development that occurs between ages 10 and 12, when brain maturation and cortical thickness in the frontal and temporal lobes peak and subcortical structures undergo synaptic pruning (13, 14). Stamm et al. found that former professional American football players, aged 40–69 years old, who began playing tackle football before age 12 exhibited greater executive dysfunction, memory impairment, and microstructural changes in the corpus callosum compared to former professional football players who began playing football at age 12 or later (15, 16). However, studies of high school and collegiate football players did not identify effects of AFE on outcomes such as the Immediate Post-Concussion and Cognitive Testing (imPACT) neurocognitive battery, Balance Error Scoring System (BESS), Brief Symptom Inventory-18 (BSI), and Wechsler Test of Adult Reading and Intelligence Scale (17–19).

In 2015, US Soccer adopted a ban on soccer heading for players aged 10 years old and younger (20), despite a lack of evidence that initiating heading before age 10 led to poorer outcomes. Caccese et al., used age 10 to retrospectively dichotomize a group of collegiate soccer players into those who began heading the soccer ball at age 10 or younger and those who began heading after age 10 (21). Caccese et al. found no difference in sensory reweighting for upright stance, a marker of sensory reliance for vestibular function, between the soccer players who began heading before or after age 10. This is the only published study to focus specifically on AFE to soccer heading. While the AFE literature has focused on microstructural, cognitive, and behavioral outcomes in American football players, these outcomes have never been studied in the context of AFE to soccer heading.

The goal of the current study is to assess whether AFE to heading at ≤ 10 years old is associated with adverse effects on brain microstructure, cognitive function, and behavioral symptoms among current adult amateur soccer players.

## 2. Methods

### 2.1. Participants

All study procedures were approved by the Institutional Review Board of the Albert Einstein College of Medicine and complied with The Health Insurance Portability and Accountability Act. Eighteen to fifty-five-year-old amateur soccer players were eligible to participate in this study if they played soccer for more than 5 years, played soccer more than 6 months per year at time of enrollment, and were fluent in English. Exclusion criteria included schizophrenia, bipolar disorder, known neurological or neurodevelopmental disorder, or illicit drug use within 30 days prior to study assessments. Study procedures have been reported in detail (22–27). In brief, soccer players were recruited by print and Internet advertisements through soccer clubs, leagues, and colleges in and around New York City. Interested individuals completed screening questions online and were contacted by study personnel to confirm eligibility and assess willingness to participate. At the study visit, participants completed (1) written informed consent, (2) a web-based demographic questionnaire, (3) the Edinburgh Handedness Inventory (28), (4) computer-based cognitive assessments (Cogstate^Ⓡ^), (5) The National Institute of Health (NIH) Patient Reported Outcome Measurement Information System (PROMIS), (6) a questionnaire regarding age of first exposure to soccer heading and primary player position (forward, midfield, defense, or goalkeeper), and (7) a Diffusion Tensor MRI (with derived DTI and NODDI measures). Study participants completed additional follow up visits related to other aims of the study. DTI, NODDI, Cogstate^Ⓡ^, and PROMIS outcomes collected at the initial visit were included in this analysis.

### 2.2. Exposure assessment

AFE to soccer heading served as the exposure of interest in this analysis. Participants were asked, “At what age did you first start heading the ball in soccer? Enter “0” if you do not usually head the ball.” If participants did not enter an answer for the AFE question on the questionnaire at the initial visit, the AFE values reported at subsequent follow-up visits (see above; not otherwise part of this analysis) were averaged and that value was taken as AFE to heading. Eighty-eight percent of participants demonstrated consistent categorization of AFE (≤ 10 years vs. >10) across all study visits. For the remaining 12% of participants who had varying categorization among study visits, 63% reported AFE at their initial visit which was representative of the majority of their responses regarding AFE across study visits. Therefore, 95.6% of participants either consistently reported AFE across study visits or had a representative AFE reported at the initial visit. In addition, test-retest reliability was calculated for AFE as both a numerical and categorical variable (with cut-off at age 10). For AFE as a numerical measure, the intraclass correlation coefficient (ICC), a measure of test-retest reliability, was estimated to be 0.87 using the SAS Proc Mixed procedure with AFE from multiple time points (ranging from 2 to 7 depending on the participant). For AFE as a binary measure (< 10 vs. > 10), the test-retest reliability is 0.73 using the SAS Proc Genmod Procedure. Whether AFE is treated as a numerical or binary variable, the measure over multiple time points exemplifies a high degree of test-retest reliability (29).

### 2.3. Magnetic resonance imaging

Imaging was performed using a 3.0T Philips Achieva TX scanner (Philips Medical Systems, Best, The Netherlands) and a 32-channel head coil. T1-weighted 3D magnetization-prepared rapid acquisition of gradient echo imaging was performed with TR/TE/TI = 9.9/4.6/900 ms, flip angle 8°, 1 mm^3^ isotropic resolution, 240 × 188 × 220 matrix. Diffusion tensor imaging was performed using 2D single-shot EPI with 32 diffusion encoding directions, b-value = 800 s/mm^2^, TR = 10 s, TE = 65 ms, 2 mm^3^ isotropic resolution, 128 × 120 matrix, 70 slices. NODDI imaging was performed using the same parameters except TE = 88 ms and three shells instead of one (6 directions at b = 300 s/mm^2^, 32 at b = 800 s/mm^2^, and 60 at b = 2,000 s/mm^2^). An auxiliary field map scan was acquired using 250 mm FOV, 3 mm isotropic resolution, TR = 26 ms, TE/ΔTE= 2.5/2.3ms, α = 26° and SENSE factor = 2 (anterior-posterior) × 2 (head-foot). The auxiliary field map was used to correct small susceptibility-induced distortions in T1-weighted and larger distortions in echo-planar images.

### 2.4. Image processing

All images were reviewed by a board-certified neuroradiologist (MLL) for structural abnormalities or evidence of prior trauma, including hemorrhage. Raw and processed images were examined by trained reviewers for image quality, artifacts, and aberrant results of processing. Images that were flagged as suboptimal were excluded from the analysis.

Image processing was performed using a high-performance computing system running the Community Enterprise Operating System (CentOS) Linux distribution. Brain extraction was performed using FreeSurfer (30) for T1W images and the Brain Extraction Tool (BET) from the FSL package (31) for field map images [FSL (RRID:SCR_002823)]. All brain extractions were reviewed and manually corrected as needed.

Diffusion data were corrected for head motion and eddy current effects using FSL, with the b = 0 s/mm^2^ image as the target volume. Brain extraction was performed by transferring the already delineated and manually inspected T1W image, first distorting it to match EPI distortions of the diffusion scan based on the field map image. Diffusion parameters including Fractional Anisotropy (FA), Radial Diffusivity (RD), Axial Diffusivity (AD), and Mean Diffusivity (MD) were obtained using DTIfit from FSL. These maps were corrected for EPI distortions and registered to the T1W volume using a linear transformation performed with FSL FLIRT. The same processing approach was employed for NODDI data, except maps of orientation dispersion (ODI), intracellular volume fraction (ICVF) and isotropic diffusion (ISO) were obtained using AMICO (32).

Imaging data from 110 healthy controls was prepared using the same preprocessing steps described above. Healthy controls consisted of non-athletes ages 18–50 years old. Exclusion criteria for healthy controls included a history of a head injury, neurological or neurodevelopmental disorder, psychiatric disorder (schizophrenia, bipolar disorder, anxiety, depression, substance use disorder), prior tobacco use, diabetes, hypertension, heart disease, or contraindication to MRI.

Data from each soccer player was compared to the set of healthy controls using Subject-based registration (SURE-Quant). This method permits identification of spatial clusters of abnormalities by comparing each individual subject to a group of normative controls. It entails mapping parameter (e.g., FA) images from each control participant to each individual soccer player using ANTs, the approach which has been shown to minimize potential registration errors (33–37). A voxel-wise analysis of covariance (VANCOVA) of the player's data against that of the control group was performed to identify subject-specific clusters of abnormalities within white matter. Statistical Type I error was controlled to below 0.01 by retaining clusters comprising at least 100 voxels that were all significant at *p* ≤ 0.01(26, 37). All imaging analyses were adjusted for effects of age and sex.

For each soccer player, we extracted total volume (mm^3^) of abnormally low and abnormally high FA, RD, AD, and MD as well as ODI, ICVF and ISO across the whole brain white matter. This resulted in 14 summary measures from each player for subsequent statistical analysis. This voxel-wise analysis method ultimately provides measures of overall abnormal microstructure burden for each participant.

### 2.5. Cognitive function

Cognitive function was assessed using subtests selected from Cogstate^Ⓡ^, a computerized, reliable and valid battery of neuropsychological tests (38, 39). Subtest selection was driven by study hypotheses. Working memory was assessed with the Two Back Test (TWOB), which measured how accurately (arcsine of square root of proportion of correct responses) participants determined if a playing card was the same as the card shown two cards previously. Processing speed was assessed with the Groton Maze chase test (GMCT), which measured how quickly and accurately (total number of correct moves per second) participants chased a target through a maze. The International Shopping List—Immediate (ISL) and Delayed Recall (ISLR) tasks measured verbal learning and memory abilities, respectively. Participants were asked to remember a list of 12 words on three consecutive learning trials and then to recall the list following a delay period. Number of correct responses was the primary outcome variable for ISL and ISLR. It is well-established that examination of cognitive functions following an insult to brain integrity, such as trauma, should be undertaken in the context of pre-morbid cognitive ability. One approach for estimating pre-injury cognitive ability is through administration of a measure of reading achievement, a “hold” ability that is resistant to the effects of brain injury (40–42). For this purpose, we administered the Word Reading subtest of the Wide Range Achievement Test-4 (WRAT).

### 2.6. Patient reported outcomes

The PROMIS short forms for depression, anxiety, sleep disturbance, anger and satisfaction in social role were utilized. These questionnaires capture participants characterizations in the previous seven days. The short forms consist of 8–12 questions from the full PROMIS questionnaire that capture the construct without sacrificing precision. PROMIS scores are reported as T-scores relative to the US population mean. A higher score indicates more of the construct being measured (24, 43).

### 2.7. Statistical analysis

Analyses were performed in R version 3.6.1 (44). AFE to soccer heading, the exposure variable of interest, was treated as a dichotomous variable with a cut-off at age 10 (AFE ≤ 10 years old and AFE >10 years old). Age 10 was used as the cut-off to investigate the US Youth Soccer rule that delays heading exposure until after age 10 (21). This divides the sample into those who began heading at age 10 or younger and those who began heading after age 10. For each category of outcomes (DTI, NODDI, Cogstate^Ⓡ^ or PROMIS), we fit a multivariate linear regression model Y_*n*×*d*_ = X_*n*×(*p*+1)_B_(*p*+1) × *d*_ + E_*n*×*d*_, where each row of Y is a vector of *d* outcome metrics of a subject (For example, volumes of abnormally high and low FA, MD, RD, AD in the DTI model), and each column of Y is a vector of *n* outcomes of a single metric, and each row of X is a vector of *p* explanatory variables (Sex, Education, Duration of Heading Exposure, Age of First Exposure Category) with 1 as the first entry, and the *b*_*ij*_ of B is the coefficient of i*th* explanatory variable and j*th* metric, with *b*_0*j*_ be the intercept term for j*th* metric, and the i*th* row of E, *e*_*i*_ is a vector of d error terms for the i*th* subject. We assume that Cov(*e*_*i*_, *e*_*j*_) = 0 for i≠j, and *e*_*i*_ follows a multivariate normal distribution MVN(0,Σ), with no restriction on the covariance matrix Σ. To obtain the maximum likelihood estimates of B and Σ in the multivariate linear regression model, we used the “gls” function in the “nlme” package in R (45, 46). The correlation structure in the regression model was specified to account for correlation among the outcome variables within each domain for each subject. This allows us to understand the effect of AFE to soccer heading on each of the continuous outcome variables, with duration of heading exposure, sex, and education as co-variates, while accounting for associations among an individual's outcome measures. Therefore, the effect of AFE group on each outcome variable is determined independent of covariates and associated outcomes. Duration of heading exposure was calculated (age—age of first exposure to heading). Age was not included as an additional covariate because this would present direct collinearity with duration. WRAT, a measure of pre-morbid cognitive ability, was used as an additional covariate in Cogstate^Ⓡ^ models. The use of these models results in lower Type 1 error and accounts for collinearity between the outcome variables within a domain for each participant by using a specified symmetric correlation structure (47). It is important to account for the associations among outcome variables within each domain, as they are not necessarily independent (47). For example, the DTI measurements, FA, RD, MD, and AD are interrelated and may all be affected by the same underlying pathological process. ANOVA was used to compare models that did or did not include AFE to heading. Models for each category of outcomes, stratified by sex, were also created. R code will be available from the authors upon request.

## 3. Results

Two hundred and seventy-six amateur soccer players were included in this analysis. The sample characteristics are presented in Table 1. Players who began heading after age 10 were, on average, older (*p* = 0.04), had more years of education (*p* = 0.04) at the time of study enrollment, and were more likely to be female (*p* = 0.05). In addition, players who began heading after age 10 had a shorter duration of heading exposure (*p* = 0.002). Proportion of participants with history of concussion, WRAT score, and handedness were similar across AFE groups. The distribution of primary player position between AFE groups was not significantly different. Descriptive statistics for the outcomes of interest are presented in Supplementary Table 1. A summary of the results of the multivariable model for each category of outcomes (Cogstate^Ⓡ^, PROMIS, DTI, NODDI) is presented in Table 2. We found that AFE ≤ 10 (i.e., earlier exposure to heading), was associated with better performance on tests of working memory (*p* = 0.03) and verbal learning (*p* = 0.02). In addition, the mixed effects model testing Cogstate^Ⓡ^ outcomes which included AFE, yielded a significantly better model fit than those without AFE (*p* = 0.03). We found no significant association of AFE to soccer heading (dichotomized at age 10) with verbal memory, visuospatial processing speed, any PROMIS, DTI, or NODDI measure in this sample. Subsequent sex-stratified analyses revealed that AFE to heading after age 10 was associated with greater depressive symptoms among men (*n* = 196, *p* = 0.01), but not women (*n* = 81, *p* = 0.13). No significant association of AFE was observed with any of the Cogstate, PROMIS, DTI, or NODDI measures in the female only sample.

**Table 1:** Player characteristics.

	**Sample (*****n*** = **276)**		**AFE** ≤ **10 (*****n*** = **212)**		**AFE** > **10 (*****n*** = **64)**		
	**Mean or** ***n***	**SD or %**	**Mean or** ***n***	**SD or %**	**Mean or** ***n***	**SD or %**	***p*** **-value**
**Demographics**							
Age (years)	25.81	7.66	25.15	6.62	28.02	10.13	**0.04**
Duration of heading (years)	17.38	7.96	18.36	7.01	14.16	9.91	**0.002**
Education (years)	15.71	2.24	15.54	2.10	16.27	2.60	**0.04**
AFE (years)	8.43	3.86	6.79	2.03	13.86	3.50	**< 0.001**
Females*	81	29.35%	56	26.42%	25	39.06%	0.05
History of concussion^*^	103	37.32%	81	38.21%	22	34.38%	0.58
WRAT	105	14.75	104.3	14.81	107.1	14.44	0.18
Laterality index	0.79	0.40	0.8	0.36	0.73	0.50	0.26

Sample characteristics, grouped by those who began soccer heading at age 10 or younger, and those who began soccer heading after age 10. Duration of heading exposure was calculated by age—AFE. Laterality Index represents handedness from −1 (left hand dominant) to 1 (right hand dominant) as determined by the Edinburgh Handedness Inventory. Mean and SD or, when denoted by an asterisk (^*^), n and % are presented. P-value represents the results of a two tailed heteroscedastic t-test comparing the exposure groups. P-values < 0.05 are highlighted in bold.

**Table 2:** Linear mixed effects models results summary.

**Variable**	**Est**.	**SEM**	**95% CI**	***p*-value**
**Cognitive performance (Cogstate** ^Ⓡ^ **)**				***0.03***
Working memory	−0.09	0.04	−0.17, −0.01	**0.03**
Psychomotor speed	−0.09	0.07	−0.23, 0.05	0.18
Verbal learning	−1.25	0.54	−2.31, −0.19	**0.02**
Verbal memory	−0.24	0.25	−0.73, 0.25	0.22
**Patient-reported outcomes (PROMIS)**				*0.48*
Anger	0.83	1.04	−1.21, 2.86	0.43
Anxiety	0.53	0.97	−1.36, 2.43	0.58
Depression	1.00	0.89	−0.74, 2.75	0.26
Sleep disturbance	−0.99	1.25	−3.43, 1.46	0.43
Satisfaction with social role	−1.19	1.00	−3.14, 0.77	0.24
**Imaging**				
*DTI*				*0.58*
High RD	20.94	286.87	−541.32, 583.21	0.94
Low RD	−94.39	262.43	−608.74, 419.96	0.72
High FA	−107.51	183.77	−467.69, 252.67	0.56
Low FA	−35.04	46.04	−125.28, 55.19	0.45
High MD	90.66	422.27	−736.98, 918.31	0.83
Low MD	−65.98	385.53	−821.64, 689.68	0.86
High AD	−205.31	294.52	−782.58, 371.95	0.49
Low AD	−84.83	192.40	−461.94, 292.28	0.66
***NODDI***				*0.93*
High ICVF	7.39	689.55	−1344.12, 1358.90	0.99
Low ICVF	−112.78	429.661	−954.92, 729.35	0.79
High ISO	−329.18	1982.45	−4214.77, 3556.41	0.87
Low ISO	42.16	41.85	−39.86, 124.18	0.31
High ODI	−175.67	604.25	−1360.01, 1008.66	0.77
Low ODI	−108.99	126.48	−356.90, 138.91	0.39

The results of the four multivariable linear models are summarized. Each category of outcomes (italicized items in the column “Variable”; Cogstate^Ⓡ^, PROMIS, DTI, and NODDI) represents a separate model that includes all outcomes within that category for each participant. Estimate, Standard Error of the Mean, 95% Confidence Interval, and *p*-value are presented for the association of AFE with each outcome. AFE ≤ 10 is the default group in each model and the sign of the estimate demonstrates the change in outcome when switching from AFE ≤ 10 to AFE > 10. The overall *p*-value for each model, determined by the log likelihood test, is reported to demonstrate whether the model including AFE is significantly better than the model without AFE. The following covariates were included in all models: (1) duration of heading exposure, (2) sex, and (3) education. The model assessing cognitive function also included the WRAT score as a covariate. *p*-values < 0.05 are highlighted in bold.

## 4. Discussion

Our analysis of a large prospectively enrolled sample of adult amateur soccer players revealed that those who began soccer heading at age 10 or younger, performed better on tests of working memory and verbal learning compared to those who began heading at an older age, while controlling for duration of soccer heading, education, sex, and verbal intelligence. We did not find evidence of a significant association of AFE to heading with adult brain microstructure or behavior. Studies of American football have suggested that AFE to football, a putative marker for onset of and thereby total lifetime exposure to RHI, confers risk for adverse neurological outcomes. However, notwithstanding recent rule changes that proscribe heading for players 10 and younger, knowledge regarding the potential association of AFE to soccer heading with adverse effects on brain structure and function is quite limited. To our knowledge, this is the first study to investigate AFE to soccer heading with respect to brain structure and cognitive function. In addition, this is the first study to demonstrate better performance on tests of cognition in those with earlier exposure to contact sports.

Our findings contribute to the growing body of knowledge on the association of early exposure to RHI with neurological outcomes later in life. However, it is important to consider how our study differs from other investigations of AFE to RHI. Studies of American football first introduced the concept of AFE to RHI as an exposure metric that might predict adverse neurological outcomes (15, 16). These studies analyzed samples of retired professional football players who were over 40 years old and reported that commencing football play before age 12 was associated with lower FA and higher RD in the corpus callosum, consistent with demyelination, smaller thalamic volume, and worse performance on tests of executive function, memory, and verbal IQ later in life (15, 16, 47, 48). However, in line with our present findings, subsequent studies failed to replicate these findings when examining former and active high school and college level American football players as well as other contact sports athletes (e.g., lacrosse, rugby, and ice hockey) (17, 18, 49–52). Notably, these subsequent studies assessed participants at a much younger age compared to the original studies of former professional American football players. In addition, the studies of younger players tested clinical outcome measures, such as imPACT, which may be less sensitive to changes in brain function compared to the assessments of former professional American football players. Compared to most reports on the role of AFE to RHI, we studied a larger sample [*n* = 30 in Caccese et al.'s study of collegiate soccer players (21)], included both men and women, and tested a broader set of outcomes, including multiple measures of brain microstructure, a comprehensive cognitive battery, and patient reported behavioral outcomes. In addition, our sample is, on average, older than previous studies of high school and college players, but younger than previous studies of retired American football players and professional fighters (14, 17, 18, 47, 49, 51, 53, 54).

Only one prior study has examined effects of AFE to heading in soccer (21). Caccese et al. tested a physiologic outcome, sensory reweighting for upright stance, a measure of vestibular function, comparing college soccer players who began heading at age 10 or earlier with those who began heading after age 10. This study did not assess cognitive or behavioral outcomes, or brain structure. Caccese et al. used a cutoff at age 10 in line with recent (at the time) US Soccer rule changes, which proscribe heading prior to age 10. All participants in our study began soccer play and heading before restrictions were promulgated for youth soccer. We tested cutoffs at both 10 years, to explore the potential impact of the new heading restrictions and tested 12 years, which was the basis of prior studies that reported an adverse association of AFE with outcomes. Twelve years old had been used in prior studies as a cutoff due to a crucial period of brain development and synaptic pruning thought to occur from ages 10–12 (13, 14). In addition, we tested a cutoff of 8 years old, the median AFE to heading in our sample, as well as treated AFE as a continuous variable. In these sensitivity analyses, we found that those who began heading before age 8, performed better on a test of working memory (*p* = 0.046) and exhibited lower volumes of low ISO (*p* = 0.028) compared to those who began heading later (see Table 3). No other associations of AFE to heading with cognitive performance, behavioral outcomes, or brain microstructure were observed in the analyses using cutoffs at age 8, age 12, or treating AFE as a continuous variable.

**Table 3:** Sensitivity analyses.

	**AFE dichotomized (AFE**<**12 and AFE** > **12)**				**AFE dichotomized (AFE**<**8 and AFE** > **8)**				**AFE continuous**			
**Variable**	**Est**.	**SEM**	**95% CI**	***p*-value**	**Est**.	**SEM**	**95% CI**	***p*-value**	**Est**.	**SEM**	**95% CI**	***p*-value**
**Cognitive performance (Cogstate** ^Ⓡ^ **)**												
Working memory	0.05	0.05	−0.04, 0.14	0.27	0.08	0.04	0.00, 0.15	**0.046**	−0.01	0.01	−0.02, 0.00	0.13
Psychomotor speed	0.07	0.07	−0.05, 0.19	0.36	0.07	0.06	−0.05, 0.19	0.23	−0.01	0.01	−0.03, 0.01	0.22
Verbal learning	1.00	0.57	−0.11, 2.11	0.08	0.33	0.48	−0.62, 1.27	0.5	−0.11	0.06	−0.23, 0.02	0.09
Verbal memory	0.24	0.27	−0.28, 0.76	0.37	−0.10	0.23	−0.54, 0.34	0.67	−0.01	0.03	−0.07, 0.05	0.78
**Patient-reported outcomes (PROMIS)**												
Anger	−0.40	1.09	−2.53, 1.73	0.71	0.13	0.92	−1.68, 1.94	0.89	−0.06	0.11	−0.28, 0.16	0.57
Anxiety	−0.36	1.01	−2.34, 1.62	0.72	−0.05	0.86	−1.73, 1.64	0.96	0.05	0.10	−0.15, 0.26	0.61
Depression	−0.56	0.93	−2.39, 1.27	0.55	−0.02	0.79	0.98, −1.58	0.98	0.09	0.10	−0.10, 0.28	0.34
Sleep disturbance	1.01	1.31	−1.55, 3.57	0.44	1.75	1.11	−0.42, 3.92	0.11	−0.03	0.13	−0.29, 0.24	0.84
Satisfaction with social role	0.40	1.05	−1.66, 2.45	0.70	0.55	0.89	−1.19, 2.30	0.54	−0.09	0.11	−0.30, 0.12	0.42
**Imaging**												
***DTI***												
High RD	20.24	300.27	−568.30, 608.77	0.95	120.51	254.94	−397.17, 620.20	0.64	−7.92	30.74	−68.17, 52.33	0.80
Low RD	107.33	274.67	−431.03, 645.69	0.70	191.87	233.08	−264.96, 648.70	0.41	−4.83	28.10	−59.91, 50.25	0.86
High FA	128.62	192.31	−248.32, 505.55	0.50	96.30	163.34	−223.93, 416.52	0.56	9.94	19.71	−28.68, 48.57	0.61
Low FA	17.57	48.23	−76.97, 112.10	0.72	−17.27	40.96	−97.55, 63.02	0.67	−1.85	4.94	−11.62, 7.83	0.71
High MD	−46.50	442.03	−912.88, 819.87	0.92	127.76	375.38	−607.98, 863.49	0.73	−10.92	45.25	−99.60, 77.77	0.81
Low MD	19.20	403.57	−771.80, 810.20	0.96	265.85	342.41	−405.27, 936.96	0.44	−17.60	41.24	−98.42, 63.22	0.67
High AD	240.04	308.22	−364.06, 844.14	0.44	178.22	261.86	−335.03, 691.46	0.50	−5.26	31.59	−67.16, 56.65	0.87
Low AD	37.87	201.45	−356.98, 432.71	0.85	171.18	170.80	−163.60, 505.95	0.32	−15.66	20.57	−55.97, 24.65	0.45
***NODDI***												
High ICVF	−169.00	721.70	−1583.52, 1245.53	0.81	−471.11	612.40	−1671.41, 729.19	0.44	7.74	73.81	−136.92, 152.39	0.92
Low ICVF	300.92	449.43	−579.95, 1181.79	0.50	62.93	382.03	−685.85, 811.71	0.87	30.17	46.03	−60.05, 120.40	0.51
High ISO	−17.31	2075.20	−4084.70, 4050.08	0.99	1070.14	1761.43	−2382.26, 4522.53	0.54	−164.92	212.31	−581.04, 251.20	0.44
Low ISO	−46.92	43.79	−132.75, 38.91	0.28	−81.34	36.94	−153.75, −8.93	**0.03**	3.64	4.42	−5.02, 12.31	0.41
High ODI	133.70	632.54	−1106.08, 1373.48	0.83	446.14	536.63	−605.64, 1497.93	0.41	−24.70	64.76	−151.64, 102.24	0.70
Low ODI	124.58	132.36	−134.84, 384.00	0.35	−61.52	112.54	−282.10, 159.07	0.58	2.81	13.56	−23.77, 2 9.39	0.84

The results of the four multivariable linear models are summarized for each of the three sensitivity analyses with differing age cut-offs. Each category of outcomes, Cogstate^Ⓡ^, PROMIS, DTI, and NODDI, represents a separate model that accounts for associations between the outcomes within that category for each participant. AFE > 12 and AFE > 8 serve as the reference group. Estimate, Standard Error of the Mean, 95% Confidence Interval, and *p*-value (bolded *p* < 0.05) are presented for the association of AFE with each outcome.

Recent heading restrictions for youth soccer, while well-intended, are not evidence-based. Motivation for the restrictions includes concern that early exposure to RHI could adversely impact a critical period in brain development. On the other hand, it is well-known that the developing brain exhibits remarkable ability to recover from injury (55). Our failure to confirm an adverse association of AFE to heading in this large sample of young to middle age soccer players could have several interpretations. Although prior adverse associations of longer-term heading with structural and functional brain outcomes have been confirmed in this cohort, AFE to heading may not be a robust indicator of long-term RHI exposure (25, 26). This could diverge from the context of collision-related RHI in American football. Heading is a skill that must be learned and integrated into a player's repertoire, potentially over a longer period, heading in youth soccer is less common (56) and, given lower ball velocity in youth play, may pose less risk for adverse effects than heading by older players. In American tackle football impacts increase in both frequency and magnitude with level of play. However, RHI in American football also begins with participation and is unavoidable (57). It is also possible that adverse outcomes are associated with AFE to heading, but may not become measurable until later in life.

While the motivation to study AFE to contact sports has focused on risk for poorer outcomes later in life, we found evidence of an opposite effect. Those who began heading at age 10 or younger exhibited better performance on tests of working memory and verbal learning as young adults, compared to players who started heading after age 10. Several studies have demonstrated that participation in physical activity in childhood is associated with better cognitive function in midlife and adulthood (58, 59). In addition, heading is a learned skill requiring balance and coordination. Better hand-eye coordination has been associated with better performance on higher-level cognitive processes, such as mathematics, independent of executive function (60, 61). This has been attributed to augmented pre-frontal cortex development (62). While RHI during childhood may be associated with poorer outcomes in adulthood, as seen in the studies of former American football players, the RHI from heading in young soccer players may not produce the same effect. In contrast, the physical activity and refinement of coordination and balance during a developmentally critical period may be associated with better cognitive outcomes in soccer players. In addition, heading in youth soccer is less frequent and entails lower magnitude impacts, compared to adult soccer and to RHI in other contact sports. In a 2013 study of a non-overlapping sample of soccer players, adverse effects on cognition and brain microstructure were found above a threshold level of cumulative heading exposure (26). Taken together, these findings indicate soccer play and heading in childhood, and soccer participation in general, may be neutral or even beneficial. However, above a certain threshold of cumulative RHI exposure in adults, beneficial effects are counterbalanced by cumulative adverse effects of RHI. This is an important area for future study.

The literature on AFE to contact sport has focused American football, which is played almost exclusively by boys and men. Soccer, however, is played by large numbers of girls and women. We have previously reported sex-divergent excess risk for brain abnormalities related to heading (27), and women are known to be at excess risk for poorer outcomes following head injury compared to men (63, 64). An important feature of our study is its identification of a differential association of AFE to soccer heading with depressive symptoms in men and women. These preliminary findings should motivate expanded inclusion of women in studies of RHI, and of AFE to contact sports, to understand RHI risk for all athletes.

Our findings must be considered in light of several limitations. AFE to heading in this study is a self-report measure and therefore could be affected by recall bias resulting in miscategorization bias if participants' reported AFE to heading is not aligned with actual exposure onset. However, given high rate of consistent reporting of AFE to heading at multiple time points, we can be more confident that the self-report AFE to heading is reliable. In addition, this sample is limited to active soccer athletes. Athletes who sustained adverse effects of RHI may have ceased soccer play and would not be captured in this study. Sex, duration of heading exposure, education, and WRAT were used as covariates, however additional confounding due to variables not identified is possible. While player position did not differ between the AFE groups in this analysis and therefore was not included as a covariate, future studies should consider the role of player position in modifying the effects of RHI. In addition, duration of soccer heading, calculated as the difference between current age and AFE to heading, assumes that participants headed continuously from when they began heading to the start of the study. We employed whole brain measures of microstructural abnormality, which minimizes the chance our findings could be insensitive or biased if we had chosen to study only specific tracts or regions *a priori*. Moreover, the same global measurement approach has identified adverse associations of microstructure with exposure to heading [e.g., (26)]. However, previous studies of AFE to contact sports and brain microstructure utilized a tract based or region of interest approach (16, 48). Our cross-sectional study design strictly precludes direct causal inference. Future investigations that study the effect of age of first exposure on objective measures of brain structure and function over time are needed to characterize persistent long-term effects. In addition, we studied an adult amateur soccer population from a single region in the United States and our findings may not generalize to other ages and levels of soccer play. However, the mean activity level of study participants has been deemed comparable to that of amateur soccer players worldwide (65). Longitudinal studies of soccer players across the lifespan will be required to better understand what exposure metrics and risk factors are most strongly associated with adverse neurological outcomes later in life. This is critical to designing approaches to educate and protect athletes of all ages.

In conclusion, we found that soccer players who began heading at ≤ 10 years old demonstrated better performance on tests of working memory and verbal learning as young adults compared to soccer players who began heading at a later age. We found no differences for a range of behavioral and microstructural measures. Future studies, including follow-up into later life and balanced inclusion of women, are required to fully understand the potential of early heading exposure to confer risk for adverse outcome, or conversely, benefit in adulthood. Further research is required to support evidence-based policy and inform individual decisions to maximize player safety.

## Data availability statement

The raw data supporting the conclusions of this article will be made available by the authors, without undue reservation.

## Ethics statement

The studies involving human participants were reviewed and approved by the Institutional Review Board of the Albert Einstein College of Medicine. The patients/participants provided their written informed consent to participate in this study.

## Author contributions

MC: conceptualization, methodology, analysis, and manuscript writing and editing. KY: methodology, analysis, and manuscript editing. RF: methodology, software, data curation, and manuscript writing and editing. BD: methodology and analysis. WS: conceptualization and manuscript editing. MZ: conceptualization, investigation, and manuscript editing. MK: analysis and manuscript editing. RL: conceptualization, manuscript editing, supervision, and funding acquisition. ML: conceptualization, investigation, manuscript writing and editing, supervision, and funding acquisition. All authors contributed to the article and approved the submitted version.
