# Supplementary material for: Age of first exposure to soccer heading: Associations with cognitive, clinical, and imaging outcomes in the Einstein Soccer Study
Source: Front Neurol. 2023 Feb 9;14:1042707. doi: 10.3389/fneur.2023.1042707 (PMC9947556; doi:10.3389/fneur.2023.1042707)
Supplement: Supplementary file 1 [file Table_1.DOCX]

# Supplementary Material

Descriptive Statistics (mean and standard deviation) for Cogstate, PROMIS, DTI and NODDI outcomes are presented and stratified by age of first exposure to soccer heading group. The p-value is the result a two-tailed heteroscedastic t-test comparing the exposure groups.

|  | **Sample (n=276)** | | **AFE <= 10 (n=212)** | | **AFE > 10 (n=64)** | |  |
| --- | --- | --- | --- | --- | --- | --- | --- |
|  | Mean | SD | Mean | SD | Mean | SD | p-value |
| ***Cogstate*** |  |  |  |  |  |  |  |
| Working Memory | 1.149 | 0.29 | 1.16 | 0.29 | 1.119 | 0.30 | 0.35 |
| Psychomotor Speed | 1.481 | 0.47 | 1.48 | 0.48 | 1.49 | 0.45 | 0.93 |
| Verbal Learning | 26.18 | 3.78 | 26.28 | 3.85 | 25.86 | 3.56 | 0.41 |
| Verbal Memory | 9.41 | 1.83 | 9.39 | 1.84 | 9.48 | 1.80 | 0.71 |
| ***PROMIS*** |  |  |  |  |  |  |  |
| Anxiety | 52.4 | 6.39 | 52.27 | 6.18 | 52.83 | 7.08 | 0.57 |
| Depression | 47.83 | 5.89 | 47.62 | 5.64 | 48.52 | 6.67 | 0.33 |
| Sleep Disturbance | 46.92 | 8.25 | 47.05 | 8.29 | 46.48 | 8.16 | 0.63 |
| Anger | 47.37 | 6.86 | 47.23 | 6.82 | 47.83 | 7.04 | 0.55 |
| Satisfaction with Social Role | 53.44 | 6.70 | 53.69 | 6.62 | 52.63 | 6.96 | 0.28 |
| ***DTI*** |  |  |  |  |  |  |  |
| High FA (mm3) | 753.2 | 1223.31 | 760.8 | 1278.25 | 728 | 1029.10 | 0.83 |
| High RD (mm3) | 945.7 | 1923.35 | 982.1 | 2043.54 | 825.1 | 1463.55 | 0.50 |
| High MD (mm3) | 1451.1 | 2838.61 | 1498.6 | 3006.58 | 1293.5 | 2205.54 | 0.55 |
| High AD (mm3) | 1197 | 1964.08 | 1259.7 | 2095.14 | 987.1 | 1441.90 | 0.24 |
| Low FA (mm3) | 144 | 304.24 | 153.3 | 308.72 | 112.9 | 289.06 | 0.34 |
| Low RD (mm3) | 742.4 | 1759.03 | 741.1 | 1924.40 | 746.4 | 1050.35 | 0.98 |
| Low MD (mm3) | 938.8 | 2573.06 | 927.2 | 2619.27 | 977.3 | 2433.12 | 0.89 |
| Low AD (mm3) | 335.1 | 1279.10 | 344.1 | 1335.36 | 305.2 | 1080.84 | 0.81 |
| ***NODDI*** |  |  |  |  |  |  |  |
| High ODI (mm3) | 634.8 | 3993.68 | 719.5 | 4527.51 | 354 | 929.23 | 0.27 |
| High ICVF (mm3) | 1772 | 4576.12 | 1863 | 5039.40 | 1472.1 | 2496.93 | 0.40 |
| High ISO (mm3) | 3436.6 | 13064.80 | 3549 | 13901.11 | 3063 | 9883.47 | 0.76 |
| Low ODI (mm3) | 591.4 | 838.04 | 600.9 | 882.54 | 560 | 675.05 | 0.69 |
| Low ICVF (mm3) | 1942.4 | 2861.94 | 1926.7 | 2839.73 | 1994.4 | 2956.57 | 0.87 |
| Low ISO (mm3) | 89.5 | 285.40 | 91.22 | 300.87 | 83.78 | 228.73 | 0.83 |
